# The characteristics of Posner-Schlossman syndrome: A comparison in the surgical outcome between *cytomegalovirus*-positive and *cytomegalovirus*-negative patients

**DOI:** 10.1097/MD.0000000000018123

**Published:** 2019-11-27

**Authors:** Kazuhiro Murata, Kyoko Ishida, Kenji Ozawa, Akira Sawada, Kiyofumi Mochizuki, Tetsuya Yamamoto

**Affiliations:** a Department of Ophthalmology, Gifu University Graduate School of Medicine; b Department of Ophthalmology, Toho University Ohashi Medical Center, Japan.

**Keywords:** *cytomegalovirus*, glaucoma surgery, Posner-Schlossman syndrome

## Abstract

This retrospective observational study aims to report the clinical characteristics and surgical results in eyes with Posner-Schlossman syndrome (PSS), and compare these outcomes between *cytomegalovirus* (CMV)-positive and -negative eyes.

We reviewed the medical records of 21 consecutive immunocompetent patients clinically diagnosed with PSS between the years 2010 and 2018. Aqueous humor was collected from all the affected eyes to detect if CMV was present, and polymerase chain reaction (PCR) was performed using the herpesvirus family primers.

The average period between the initial PSS attack and aqueous humor sampling at our institute was 9.3 years. Out of the 21 patients, 62% were CMV-positive. Regardless of CMV status, the mean intraocular pressure (IOP), mean deviation (MD), and central corneal endothelium cell (CEC) density, at the initial examination at our institute were already significantly worse in the affected eyes than in the unaffected eyes (all *P* values < .05). The average visual acuity (VA) was only significantly worse in the CMV-positive group (*P* = .02). Out of all the patients, those that were CMV-positive had undergone more glaucoma surgeries (*P* = .056). Fourteen patients underwent either a trabeculectomy (TRAB) or a trabeculotomy (LOT), and their IOP significantly reduced following surgery (*P* < .001). In 85.7% of those that had surgery, their IOP was successfully lowered to less than 20 mm Hg.

Long-lasting PSS causes a decrease in VA, MD, and the CEC density. A prompt diagnosis is required, and an appropriate treatment plan should be formulated. In those patients with PSS that develop uncontrolled glaucoma, both TRAB and LOT may be effective in controlling IOP.

## 1 Introduction

Posner-Schlossman syndrome (PSS) is a rare ocular disorder, with recurrent unilateral attacks of anterior non-granulomatous uveitis, and elevated intraocular pressure (IOP).^[1,2]^ Although believed to be a self-limiting benign disorder, it is now recognized as the cause of open angle glaucoma (OAG).^[2–4]^ The treatment for PSS is designed to control the inflammation and elevation in the IOP. However, some patients do not respond to topical steroids or anti-glaucoma medications. In these cases, IOP-lowering surgery is needed to prevent progression of the visual field defects.^[5]^

The etiology of PSS has not been definitively determined, however, it has been suggested that the herpes simplex virus (HSV) and *cytomegalovirus* (CMV) could play a role, because of the presence of their deoxyribonucleic acid (DNA) in the aqueous humor during acute PSS attacks.^[2,6,7]^ Although several studies have reported that CMV is more likely to cause PSS than HSV,^[8–13]^ to our knowledge, only 2 studies,^[13,14]^ have compared the clinical characteristics of CMV-positive PSS patients to that of CMV-negative PSS patients.

Thus, the purpose of this study is to report the clinical characteristics and surgical results in eyes with PSS and compare these outcomes between CMV-positive and CMV-negative eyes.

## 2 Methods

### 2.1 Patients

This retrospective observational study protocol was approved by the Institutional Review Board of Gifu University Graduate School of Medicine. We reviewed the medical records of consecutive immunocompetent patients, with clinically diagnosed PSS between the years 2010 and 2018 in the Department of Ophthalmology, Gifu University Graduate School of Medicine.

The clinical diagnosis of PSS was based on the clinical features of the disease described previously by Jap et al^[4]^ including mild unilateral anterior uveitis with high IOP, small white keratic precipitates (KP) on the endothelial surface of the central cornea, no posterior synechia, and no inflammatory lesions in the posterior segment of the eye. The anterior chamber (AC) inflammation in these patients with PSS, generally resolved within few weeks of the initiation of topical steroids, but anterior uveitis with a high IOP frequently reoccurred. Other etiologies of uveitis^[4,5]^ were ruled out with routine systemic investigations including: chest X-ray, a tuberculin skin test, serum angiotensin-converting enzyme test, and serological tests for syphilis, toxoplasmosis, and the human T-lymphotropic virus-1.

### 2.2 Polymerase chain reaction analysis

All procedures to be performed were fully explained to the patients, and informed consent was obtained. Aqueous humor samples of 100 μl were collected from all the patient's eyes, during glaucoma surgery or during an acute PSS attack. After 2012, when patient with clinical diagnosis of PSS showed inflammatory attack, aqueous sampling was performed at out-patient office. Also, whenever patients with clinical diagnosis of PSS underwent glaucoma surgery, aqueous sampling was performed without concurrent inflammation. Polymerase chain reaction (PCR) was performed using the herpesvirus family primers, for example, primers for HSV, varicella-zoster virus (VZV), and CMV. Until 2016, routine clinical single-PCR assays for HSV, VZV, and CMV DNA was outsourced to a major commercial laboratory (SRL Inc., Tokyo, Japan). After 2016, The Laboratory for Ocular Immunology, Kobe City Eye Hospital has performed the multiplex solid-phase strip PCR assay.^[15]^

### 2.3 Study design

The patient's clinical information was collected from their medical charts including: sex, age, laterality, best corrected visual acuity (BCVA), central corneal endothelium cell (CEC) density (Konan Specular Microscope X II; Konan, Aichi, Japan), IOP measured using a Goldmann applanation tonometer (Haag-Streit Holding AG, Koeniz, Switzerland), mean deviation (MD) obtained by the Humphrey Field Analyzer with the Central 30-2 program (Carl Zeiss, Germany), attack frequency, medical history, responsiveness to topical steroids and/or ocular anti-glaucoma therapy, surgical history, and follow-up duration. The patients who were CMV-positive and presented with persistent AC inflammation and/or uncontrolled elevated IOP, were treated with an induction dose of 450 mg of oral valganciclovir twice daily for a minimum of 4 weeks, followed by topical 0.5% ganciclovir for 6 times daily. This treatment was stopped in cases where there were severe adverse effects. All patients were treated with topical steroids and/or antiglaucoma medications when necessary. When patients did not respond to glaucoma medical therapy for IOP reduction, and had progressive visual field defects, glaucoma surgery, trabeculectomy (TRAB) with the aid of mitomyacin C or trabeculotomy (LOT) was performed.

After the data was collected, the patients were divided into 4 groups: patients with an eye that required either glaucoma surgery or were monitored on medication, and patients who had either a CMV-positive or CMV-negative aqueous humor sample.

### 2.4 Statistical analyses

Baseline characteristics were compared between the affected and contralateral, unaffected eyes. The ocular clinical characteristics were compared between the patients who required glaucoma surgery and those that were monitored on medication. The CMV-positive and CMV-negative groups were also compared. Quantitative variables were compared using either a paired or unpaired Student *t* test and numeric variables that were not normally distributed, were compared using the Mann–Whitney *U* test when appropriate. Categorical variables were compared using the Fisher exact test. A *P* value of <.05 was taken to be significant. All statistical analyses were performed using SPSS software version 16.0 (SPSS Japan, Tokyo, Japan.).

## 3 Results

### 3.1 Patients’ demographics and results of clinical examinations

The total number of patients with clinically diagnosed PSS was 21. The demographics of the 21 PSS patients, and their associated clinical findings at their initial examination at our hospital are presented in Table 1. The patients were predominantly men (71.4%). Eleven of the patients were affected in the right eye and 10 in the left. The mean age of the patients at the initial examination was 51.9 ± 15.0 years (median, 49 years). The mean age at the first PSS attack was 41.0 ± 18.2 years (median, 40 years). The mean IOP was significantly higher in the affected eyes than in the unaffected eyes (*P* < .001). The average BCVA (*P* = .044), MD (*P* < .001), and CEC density (*P* < .001) were significantly lower in the affected eyes than in the unaffected eyes.

**Table 1:**
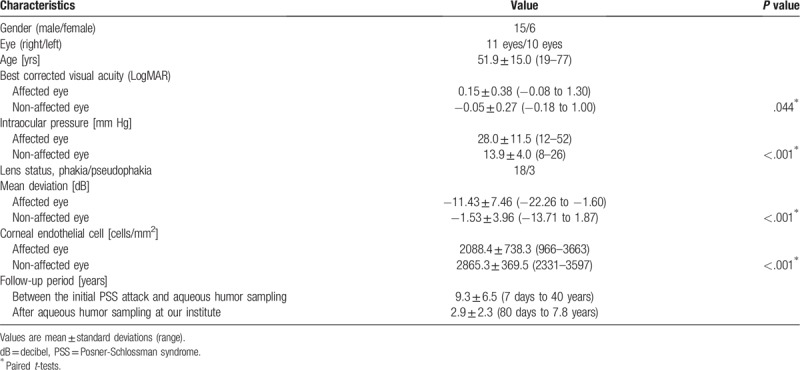
Demographic data of the patients with Posner-Schlossman syndrome.

The average period between the initial PSS attack and aqueous humor sampling was 9.3 ± 6.5 years. Eleven patients had aqueous samplings at out-patient office during acute attacks and four were CMV positive. Ten patients underwent aqueous samplings during surgery and 9 were CMV positive. The total follow-up period after aqueous humor sampling was 2.9 ± 2.3 years.

### 3.2 Comparison between patients who required glaucoma surgery and those who were monitored on medications

All the patients used topical anti-glaucoma medications, and 20 used topical steroids intermittently for their affected eyes during the follow-up period. Table 2 shows the comparisons in the clinical characteristics in the patients who did and did not require glaucoma surgery. Fourteen patients (66.7%) required glaucoma surgery. The mean IOP at the initial examination at our institute was significantly higher in the eyes of the patients who required glaucoma surgery, than in those monitored on medications (*P* = .042). Although it was statistically marginal (*P* = .056), the CMV-positive rate tended to be higher in the patients who had glaucoma surgery than in those who did not (78.6% vs 28.6%, respectively). The other clinical factors initially examined, including: BCVA, MD, and CEC density, were not significantly different between the two groups (all *P* values > .05).

**Table 2:**
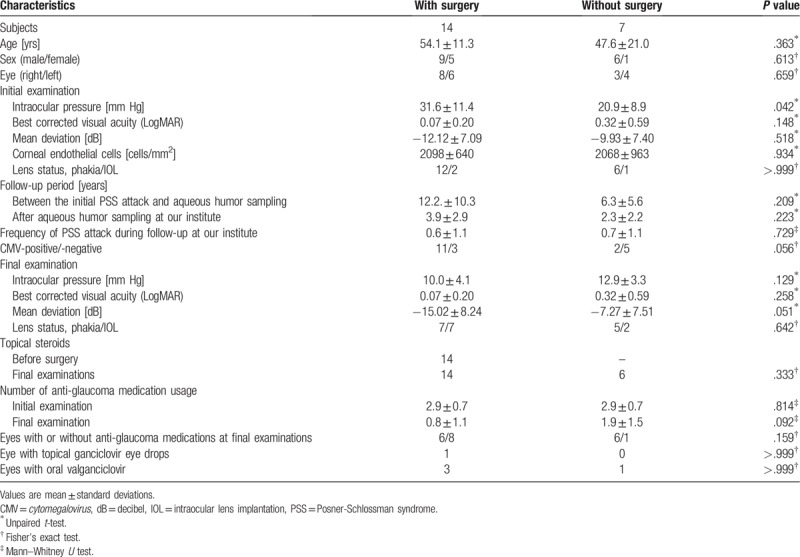
Comparisons of the clinical characteristics in patients who did or did not have glaucoma surgery.

In the surgical group, the IOP significantly reduced after surgery (*P* < .001), and the final mean IOP was 10.0 ± 4.1 mm Hg. The surgical success rate in reducing IOP to <20 mm Hg was 85.7% (12 of 14).

The MD at the final examination was marginally worse in those that had undergone glaucoma surgery, than in those who were monitored on medications (−15.02 ± 8.24 vs −7.27 ± 7.51 decibel (dB), respectively, *P* = .051). However, the difference in IOP between the two groups at the final examination was not significant (10.0 ± 4.1 vs 12.9 ± 3.3 mm Hg, respectively, *P* = .129). One patient from the medication group, and 3 patients from the surgical group had cataract surgery after the glaucoma surgery, during the follow-up period.

There was no statistical difference in the MD between the initial examination, and the final examination, in either the surgical group (*P* = .113) or the medication treatment group (*P* = .417). However, 6 patients showed a significant deterioration in MD, more than −3 dB from the baseline, at the final examinations. The reasons for this deterioration include: untreated cataract progression in 3 of the patients from the surgical group, and glaucoma progression in 2 patients from the surgical group and 1 from the medication treatment group.

There was no difference in the frequency of PSS attacks during the follow-up period at our institute between the two groups. Five (35.7%) of the 14 patients had eye inflammatory attacks after glaucoma surgery. However, 4 (80%) of the 5 patients were successful in IOP control, during inflammation.

Out of the 14 patients who had glaucoma surgical treatment, 4 underwent LOT and 2 had LOT combined with cataract surgery. The remaining 10 patients underwent TRAB with the aid of MMC. One patient after LOT and 2 patients after TRAB had further cataract surgery during the follow-up period. There was no statistical difference in baseline IOP, between the patients who underwent TRAB or LOT (*P* = .632). The final IOP measurement was not statistically significant between those that either underwent TRAB (8.9 ± 4.3 mm Hg) or LOT (12.8 ± 2.1 mm Hg) (*P* = .118). Although 2 TRAB and 4 LOT patients required additional topical anti-glaucoma medications after surgery.

The baseline MD was worse in the TRAB patients than in the LOT patients (−14.23 ± 6.67 vs −6.78 ± 5.57 dB, respectively, *P* = .072), and the final MD was significantly worse in the TRAB than in the LOT patients (−18.55 ± 6.60 vs −6.19 ± 4.26 dB, respectively, *P* = .005). There was no statistical difference between MD at the initial examination and that at the final examination both in the TRAB patients (*P* = .08) and in the LOT patients (*P* = .486). However, the MD in 5 patients significantly deteriorated, by more than −3 dB, from the baseline at the final examination. The reasons for this deterioration include: untreated cataract progression in 3 patients, and glaucoma progression in 2 patients who underwent TRAB.

### 3.3 Comparison between patients with CMV-positive and CMV-negative eyes

Among the 21 patients who underwent an aqueous humor biopsy, 13 (61.9%) were CMV-positive and 8 were CMV-negative. None of the patients were positive for HSV or VZV-DNA. In the CMV-positive group, the mean CMV immunoglobulin (Ig) G titer was 22.32 ± 13.84 units. One patient was the primary infection and CMV IgM positive (>.80). In the CMV-negative group, the mean CMV IgG titer was 13.51 ± 10.62 units, and none were CMV IgM-positive.

Thus, 3 of 4 patients detected at out-patient office during acute attacks were reactivation of attacks and 9 patients detected during surgery were chronically related.

The demographics of the CMV-positive and CMV-negative patients are presented in Table 3. There was no significant difference between the two groups with respect to age, sex distribution, laterality, or results of ophthalmic examinations such as IOP, BCVA, MD, and CEC density at the initial examination at our hospital.

**Table 3:**
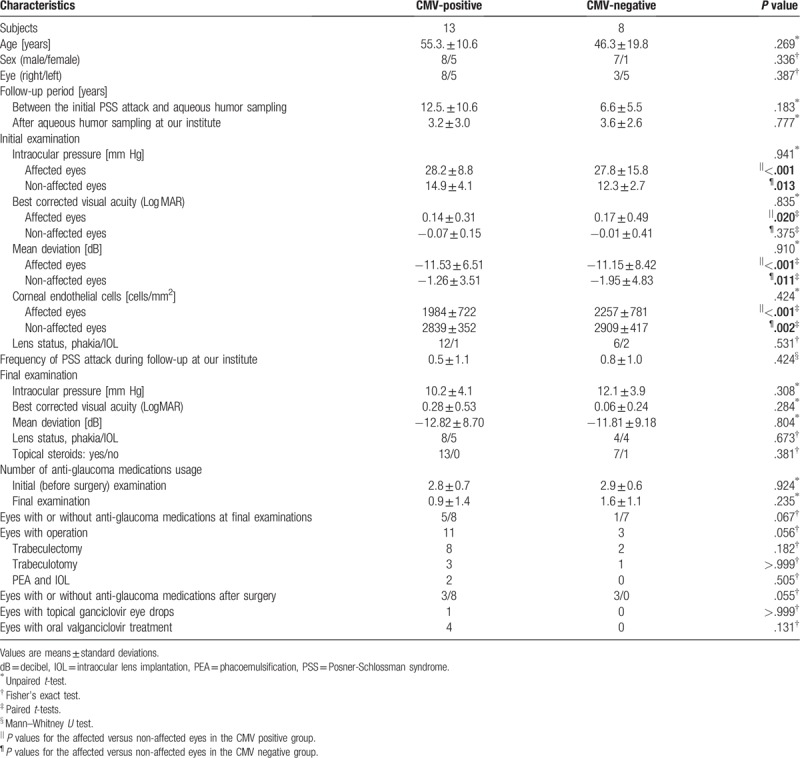
Comparisons of the clinical characteristics in patients with or without *cytomegalovirus* in the aqueous humor.

Regardless of CMV status, the mean IOP at the initial examination was significantly higher in the affected eyes than in the unaffected eyes (*P* < .001 in the CMV-positive and .013 in the CMV-negative). The mean MD (*P* < .001 in the CMV-positive and .011 in the CMV-negative) and CEC density (*P* < .001 in the CMV-positive and .002 in the CMV-negative) were significantly lower in the affected eyes than in the unaffected eyes at the initial examination. The average BCVA was significantly worse in the affected eyes than in the unaffected eyes of the CMV-positive patients (*P* = .020).

The interval between the initial PSS attack, the aqueous humor sampling, and the follow-up period was not different between the two groups. The frequency of PSS attack during the follow-up period at our institute was not statistically different between the CMV-positive and CMV-negative patients.

The requirement for glaucoma surgery was higher in the CMV-positive patients at 84.6% (11/13) than in the CMV-negative patients at 37.5% (3/8) (*P* = .056). Eight out of the 11 patients who were CMV-positive underwent TRAB, and 2 of the 3 patients who were CMV-negative underwent TRAB. The IOP of both CMV-positive and negative patients significantly decreased, and there was a significant difference in IOP between the initial and final examinations (*P* < .001 in the CMV-positive group, and *P* = .023 in the CMV-negative group). At the final examinations, the IOP (*P* = .308), the BCVA (*P* = .284), the MD (*P* = .804), and the number of anti-glaucoma medications (*P* = .924) did not differ significantly between the CMV-positive and CMV-negative patients.

Because of persistent AC inflammation and/or uncontrolled elevated IOP, 4 out of the 11 patients who were CMV-positive, were treated with topical ganciclovir, and/or oral valganciclovir. One patient with a CMV IgM-positive eye, was prescribed oral valganciclovir prior to glaucoma surgery, but there was no systemic CMV disease except for anterior uveitis. In the CMV-positive group, the 4 patients who required valganciclovir therapy, had a statistically worse CEC density, at the initial examination, than the 9 patients who did not have the anti-CMV therapy (1274.8 vs 2299.8 cells/mm^2^; *P* = .011), and the IOP was also statistically significant (37.3 vs 26.3 mm Hg, respectively, *P* = .034).

The difference in the mean MD, at the initial examination, between those treated with and without valganciclovir, was not significant (−12.82 dB vs −11.81 dB, respectively, *P* = .503).

## 4 Discussion

CMV-DNA was detected by the aqueous humor sampling in 61.9% of the Japanese PSS patients in this study. While in Singapore, a retrospective study of 67 patients with presumed PSS, reported that 52.2% were CMV-positive.^[10]^ Another prospective study noted that 26.4% (14/53) of PSS patients were positive for CMV-DNA in their aqueous humor.^[12]^ An Irish study reported that 44.4% (4/9) of PSS cases were positive for CMV-DNA in their aqueous humor.^[11]^ In mainland China, 45.0% (27/60) of PSS patients tested positive for CMV-DNA, and in Taiwan, 54% of PSS patients tested positive for CMV-DNA in their aqueous humor.^[14]^ Although the difference in the rate of endemic infection, and repeated tapping to obtain aqueous humor may affect the CMV-positive rate, its significance seems to be common globally because the control group comprised of matched patients undergoing routine cataract surgery had very low levels of CMV DNA in the aqueous.^[12,13]^

CMV has an affinity for CECs and trabecular meshwork (TM) cells.^[16]^ It has been suggested that the pathogenesis of CMV, which in the majority of cases is a latent reactivation, induces an immunologic response, that is responsible for both the reduction in the density of CECs, and the acute trabeculitis-related impairment of aqueous outflow that results in an intractable rise in the IOP.^[5]^ In our study, only 1 out of the 13 CMV-positive patients, was seropositive for anti-CMV IgM. Therefore, at the time of uveitis attack, the remaining 12 patients were suffering from a latent reactivation. One of the main causes of glaucomatous eyes, is considered to be an elevation in transforming growth factor-β (TGF-β), in the AC of glaucoma patients. TGF-β not only increases extracellular matrix production, but also inhibits its degradation.^[17]^ TGF-β also increases TM cell contraction, as well as decreasing the population of TM cells by inhibition of cell proliferation. Combined these changes contribute to the decrease in outflow of aqueous humor. An in vitro study by Choi et al^[18,19]^ reported that in CMV anterior uveitis, large amounts of TGF-β are produced early on in the infection. With repeated recurrence, the decreased cellularity of TM cells, may cause the elevation of IOP and result in a poor prognosis for patients.^[18]^ In the current study, the mean IOP was significantly higher in the affected eyes, than in the unaffected eyes. The mean BCVA, MD, and CEC density was significantly worse in the affected eyes than in the unaffected eyes at the initial examination at our institute. The mean IOP at the initial examination was significantly higher in the patients who required glaucoma surgery, than in the patients who were monitored on medication. The initial high IOP seen at our institute might have reflected the decreased cellularity of TM cells and outflow facilities, and could have necessitated the need for glaucoma surgery. CEC loss in the diseased eye, relative to the healthy eye, was significantly higher in CMV-positive PSS patients.^[14]^ The average BCVA was significantly worse in the affected eyes, than in the unaffected eyes, in the CMV-positive patients only. The average period between the initial PSS attack and aqueous humor sampling was 9.3 ± 6.5 years. These findings suggest that a prompt diagnosis, and appropriate treatment is required in PSS patients, to preserve the function of TM and CEC density, therefore, improving prognosis.

In the literature, initial therapy for CMV infection included, the systemic administration of ganciclovir and valganciclovir, and the intravitreal injection of ganciclovir.^[5,10,14,20–22]^ Topical ganciclovir and valganciclovir have also been used as an induction and a maintenance therapy in previous studies.^[5,10,14,21,23]^ The concurrent use of systemic and topical ganciclovir for a short period can reduce the ocular CMV level significantly during a recurrence of ocular hypertension.^[21]^ Mietz et al^[20]^ reported that treatment with ganciclovir led to a decrease in inflammation and normalization of the IOP. In the CMV-positive group, CEC density, and IOP was significantly worse in the 4 patients who needed valganciclovir therapy, than in the 9 patients who did not require anti-CMV therapy. We treated CMV-positive patients who presented with persistent AC inflammation and/or uncontrolled elevated IOP, with anti-CMV medication. Although, we did not determine the CMV copy numbers in the aqueous humor, these eyes needed anti-CMV medication, and so might have had a high CMV viral load. Because, a higher CMV copy number increases the number of pressure-lowering drops required.^[22]^ Also, the higher the CMV viral load in the aqueous humor, the greater the CEC loss.^[24]^

Despite the anti-CMV therapy, 50% (2/4) had a recurrence of symptoms. In a previous study, the rate of acute CMV anterior uveitis recurrence, after systemic and intravitreal ganciclovir treatment was 84.6%. However, this rate dropped to 57.1% with continual use of topical 0.15% ganciclovir.^[10]^ Alternatively, the high concentration of 2% topical ganciclovir applied every 2 to 3 hours daily as an induction therapy, and every 4 hours as a long-term maintenance therapy, has been reported to reduce the acute CMV anterior uveitis recurrence rate, including IOP spike to 36.8%.^[14]^ The use of ganciclovir as both an induction and maintenance therapy, appears to effectively stop CMV activity. Nevertheless, despite anti-CMV therapy, IOP decompensation can still occur in PSS.^[25]^ Following viral clearance, sequential uveitis may cause an IOP spike.^[14]^

During long-term follow-ups of patients with PSS, some will develop OAG. The overall number of IOP relapses were correlated with the follow-up duration,^[26]^ and the exact incidence of OAG was not known, but could increase with longer follow-up periods. Jap et al^[4]^ found that 26% of 53 PSS eyes had glaucomatous optic nerve damage, and that patients who have had PSS for 10 years or more, were 2.8 times more likely to develop glaucoma, compared with patients who have, had the disease for less than 10 years. In a retrospective study, 50% (6/12) of PSS patients required glaucoma surgery, with a mean follow-up period of 40 months.^[27]^ CMV-positive patients treated with anti-CMV medications, with a disease duration longer than 5 years, exhibited a significantly higher probability (35%) of requiring surgery.^[14]^After anti-CMV treatment, 20.8% of positive CMV patients, with an average 4-year follow-up period, required glaucoma surgery.^[26]^ In accordance with previous studies,^[5,14]^ CMV-positive patients in the current study required more glaucoma surgeries than CMV-negative patients. The high surgery rate in the current study (66.7% of the total patients required glaucoma surgery) may be related to the long duration of PSS, and a high positive CMV rate in the aqueous humor.

TRAB has been reported to reduce the severity and recurrence rates of uveitis,^[28]^ and it may prevent future attacks.^[4,29]^ The filtering bleb created by TRAB allows aqueous humor to flow into the subconjunctival space, and it may help drain inflammatory cells from the AC, thus reducing the severity of uveitis attacks, including AC inflammatory activity, and trabeculitis.^[30]^ Five (35.7%) out of the 14 patients, had inflammatory attacks after surgery. However, 80% of the patients had their IOP successfully brought under control during KP and flare up in the current study.

On the other hands, the eyes of patients with uveitis contained more fibroblasts, lymphocytes, and macrophages in conjunctiva than in the control eyes, which increases the risk of surgical failure due to scarring.^[2]^ TRAB with MMC was less effective in maintaining a reduction in IOP, in patients with uveitic glaucomatous eyes, including those with PSS, than in OAG patients.^[31]^ Postoperative uveitis was a negative predictor for surgical success.^[27]^ In the current study, 10 patients underwent TRAB and 4 had LOT. The indication for TRAB was determined by target IOP levels. Surgery successfully lowered IOP to <20 mm Hg in 90% (9/10) of TRAB and 75% (3/4) of LOT treated patients. The final mean IOP in the TRAB patients were 10 mm Hg. Of 47 patients with uveitic glaucoma, including 6 with PSS, undergoing glaucoma surgery, success rates were 82.9% for TRAB, 62.5% for LOT, and 75% for trabectome.^[27]^ Trabectome surgery with oral ganciclovir therapy has been reported to be able to control IOP for 1 year, in 100% (7/7) of patients with PSS.^[32]^ TRAB, LOT, and trabecutome are effective methods for the management of PSS with uncontrolled IOP.^[24,32]^ However, glaucoma surgery may accelerate the progression of cataracts in patients with uveitis.^[33]^ In our study, 3 patients who underwent glaucoma surgery, also required cataract surgery, and a further 3 patients showed MD deterioration, because of untreated cataracts. Although both TRAB and LOT seem to be effective, preoperative and postoperative follow-ups should include control of inflammation and the necessary treatment for cataracts should be ensured. Recurrence of inflammatory attacks was observed in 4 (4/10) patients who had TRAB and in 1 patient (1/4) who had LOT. Further studies, with larger numbers of patients, are required to determine which surgical strategy would be more favorable to control IOP and suppress inflammatory attack in PSS patients with uncontrolled glaucoma.

There was no difference in the clinical characteristics, between the CMV-positive and the CMV-negative patients, except for the higher rate of glaucoma surgery, and a significantly worse BCVA, in the affected eyes than in the unaffected eyes in the CMV-positive patients. The etiology of PSS has not been determined, but several factors other than viral infections have been proposed as possible contributors to the development of the disease such as: autoimmune, autonomic dysregulation, allergic conditions, and vascular endothelial dysfunction.^[34]^ Further research is required to elucidate the pathology of PSS.

This study had limitations. The study design was retrospective, and the sample size was small. Secondary, all of the CMV-positive patients were not treated with anti-virus therapy. It is known that CMV can be reactivated when circulating monocytes with latent CMV are recruited to sites of inflammation.^[35]^ Inflammatory cytokines are known to play a role in this.^[36]^ PSS eyes have an elevated aqueous chemokine concentration, however, the presence of CMV-DNA is not associated with any significant changes in the type of cytokine expression in patients with PSS.^[12]^ Treatment with dexamethasone decreases TGF-β levels for the first 5 days after CMV infection.^[18]^ Thus, all patients were treated with topical anti-inflammatory steroids, for a short time during inflammatory attacks in the current study. Thirdly, the follow-up periods between the initial PSS attack and aqueous humor sampling became heterogeneous. In our hospital, aqueous sampling was routinely performed after 2012. When patient with clinical diagnosis of PSS showed inflammatory attack, aqueous sampling was performed at out-patient office. Whenever patients with clinical diagnosis of PSS underwent glaucoma surgery, aqueous sampling was performed without concurrent inflammation. There is no significant difference (12.2 ± 10.3 in patients with surgery vs 6.3 ± 5.6 years in patients without surgery, *P* = .209), however, patients with glaucoma surgeries had longer time before aqueous sampling. Although timing of aqueous sampling is heterogeneous, the positive rate of CMV-DNA in this study was very high of 61.9%, comparing to previous reports.^[10–12,14]^ Especially, high positive rate in patients with glaucoma surgeries may suggest that long-lasting latent infection make damage to TM. A large prospective follow-up study with a fixed sampling and treatment regimen is required.

In conclusion, clinicians should suspect CMV in patients who have mild unilateral anterior inflammation, with recurrences of elevated IOP, and perform an aqueous humor biopsy for PCR analysis, to detect any virus DNA. CMV-positive PSS patients required more glaucoma surgeries than CMV-negative patients. Long-lasting PSS causes a decrease in BCVA, MD, and CEC density. A prompt diagnosis and appropriate treatment is required in PSS patients to improving their prognosis. For PSS patients who develop uncontrolled glaucoma, both TRAB and LOT may be effective to control IOP.

## Author contributions

**Data curation:** Kazuhiro Murata, Kyoko Ishida, Kenji Ozawa, Akira Sawada, Kiyofumi Mochizuki.

**Formal analysis:** Kyoko Ishida, Kiyofumi Mochizuki.

**Investigation:** Kyoko Ishida.

**Methodology:** Kyoko Ishida, Kiyofumi Mochizuki.

**Project administration:** Kyoko Ishida.

**Supervision:** Tetsuya Yamamoto.

**Writing – original draft:** Kyoko Ishida, Kiyofumi Mochizuki.

**Writing – review & editing:** Kyoko Ishida, Tetsuya Yamamoto.
